# Full-length transcriptome reconstruction reveals genetic differences in hybrids of *Oryza sativa* and *Oryza punctata* with different ploidy and genome compositions

**DOI:** 10.1186/s12870-022-03502-2

**Published:** 2022-03-21

**Authors:** Wenting He, Xianhua Zhang, Pincang Lv, Wei Wang, Jie Wang, Yuchi He, Zhaojian Song, Detian Cai

**Affiliations:** 1 School of Life Sciences, Hubei University, Wuhan, 430062 People’s Republic of China; 2 State Key Laboratory of Biocatalysis and Enzyme Engineering, School of Life Sciences, Hubei University, Wuhan, 430062 People’s Republic of China; 3 Wuhan Polyploid Biotechnology Co., Ltd., Wuhan, 430345 People’s Republic of China

**Keywords:** Allotetraploid rice, Distant hybrids, Single molecular real-time sequencing, Alternative splicing, Alternative polyadenylation analysis

## Abstract

**Background:**

Allopolyploid breeding is an efficient technique for improving the low seed setting rate of autotetraploids in plant breeding and one of the most promising breeding methods. However, there have been few comprehensive studies of the posttranscriptional mechanism in allopolyploids.

**Results:**

By crossing cultivated rice (*Oryza sativa,* genome AA) with wild rice (*Oryza punctata*, genome BB), we created hybrid rice lines with different ploidy and genome compositions [diploid hybrid F01 (AB), allotetraploid hybrid F02 (AABB) and F03 (AAAB)]. The genetic differences of the hybrids and the mechanism of allopolyploid breeding dominance were revealed through morphological and cytological observations and single molecule real-time sequencing techniques. The tissues and organs of allotetraploid hybrid F02 exhibited "gigantism" and the highest levels of fertility. The numbers of non-redundant transcripts, gene loci and new isoforms in the polyploid rice lines were higher and the isoform lengths greater than those of the diploid line. Moreover, alternative splicing (AS) events occurred twice as often in the polyploid rice lines than the diploid line. During these events, intron retention dominated. Furthermore, a large number of new genes and isoforms specific to the lines of different ploidy were discovered.

**Conclusions:**

The results indicated that alternative polyadenylation (APA) and AS events contributed to the complexity and superiority of polyploids in the activity of translation regulators, nucleic acid binding transcription factor activities and the regulation of molecular function. Therefore, these APA and AS events in allopolyploid rice were found to play a role in regulation. Our study provides new germplasm for polyploid rice breeding and reveals complex regulatory mechanisms that may be related to heterosis and fertility.

**Supplementary Information:**

The online version contains supplementary material available at 10.1186/s12870-022-03502-2.

## Background

Rice is one of the most important food crops in the world and is consumed by more than half of the world’s population [1]. However, with the global population increasing and the area of arable land decreasing year on year, coupled with environmental deterioration, grain yields are no longer sufficient to meet demand, especially in Asia and Africa [2, 3]. Creating hybrid varieties is recognized as one of the most suitable methods of increasing rice production. Hybrid varieties account for over 50% of all rice grown in China [4]. Over the past few years, however, rice production has stagnated due to crop intensification and other biological and abiotic factors. Therefore, there is an urgent need to develop a new pathway to increase rice yield greatly.

Polyploidization, one of the most important evolutionary events in plants, can increase genetic diversity, introduce new genetic combinations, foster adaptation to environments, and create vigorousness effects [5–7]. A great number of crops are polyploids. Especially the main crops such as wheat, cotton, and oilseed rape, all experienced the distant hybridization and polyploidization events which resulted in doubled yields [8, 9]. Inspired by the evolution of plants, a new pathway that breeding super rice by using double advantages of distant hybridization and polyploidization was developed by Cai et al. [10]. It can be divided into three stages: utilization of heterosis between subspecies (*indica* and *japonica*), between species (cultivated rice and wild rice with A genome) and between genomes (cultivated rice and wild rice with non-A genome). An efficient technology that develops synthetic allopolyploid rice based on wide cross, embryo rescue, and in vitro colchicine treatment was established [11]. Using this technology, fourteen allopolyploid rice lines were developed from the distant hybrids between cultivated rice (*Oryza sativa*) and *O.rufipogon*, *O.barthii*, *O.nivara*, *O.longistaminata*, *O.punctata*, *O.officinalis*, *O.alta*, *O.australiensis*, *O.meyeriana* [11–14].

Hybrid and polyploidy species often undergo alternative splicing (AS) and alternative polyadenylation (APA) events to increase diversity. AS and APA are two important posttranscriptional regulatory mechanisms that can enhance the diversity of the transcriptome and, ultimately, the proteome [15, 16]. AS events generate multiple transcripts from a single gene to increase transcriptional diversity [17]. The wide prevalence of AS in eukaryotes controls a range of developmental programmers and responses to a variety of environmental conditions. RNA sequencing (RNA-seq) of rice revealed that about 40–60% of intron-containing transcripts were alternatively spliced in different tissues at various developmental stages, and this was related to developmental regulation [18]. More recently OsDR11, a rice LAMMER kinase gene, was shown to transcribe two AS transcripts, OsDR11L and OsDR11S, that play opposite roles in rice disease resistance [19]. Meanwhile, APA can generate transcript variants with different 3' ends to affect the stability and expression of transcripts, resulting in diversity and complexity in the transcriptome and proteins [20]. Over the past decade, high-throughput RNA-seq studies using second-generation sequencing (SGS) technology have been used to investigate AS and APA events [18, 21]. With advancements in sequencing technology, the Pacific BioSciences (PacBio) sequencing platform, a single-molecule sequencing technology, offers great improvements in read lengths compared with those obtained using SGS [22]. This technology has been widely used in the study of AS and APA events in plants [23–26]. However, few AS and APA events have been explored in rice using PacBio sequencing technology [27].

In this study, distant hybrids with different ploidy and genome compositions were investigated. A diploid hybrid F01 (AB, 2n = 2x = 24) was produced from *O. sativa* × *O. punctata*; allotetraploid hybrid (F02, AABB) was produced through chromosome doubling of F01; and allotetraploid hybrid (F03, AAAB) was obtained by crossing tetraploid *O. sativa* (AAAA) × F02 (AABB). The differences in fertility and morphology of the hybrids were studied using morphology and cytology. Moreover, the full-length transcriptomes of the hybrids were analyzed comprehensively for the first time. AS and APA events in the different hybrids were globally surveyed using PacBio single-molecule long-read isoform sequencing, and the regulatory complexity of allotetraploid rice was explored through a variety of database annotations. This study lays a foundation for the analysis of the molecular mechanism of allopolyploid breeding dominance and provides a useful reference for rice transcriptome annotation resource and genetic diversity.

## Results

### Morphological and agronomic characteristics with different ploidy levels

**Fig. 1:**
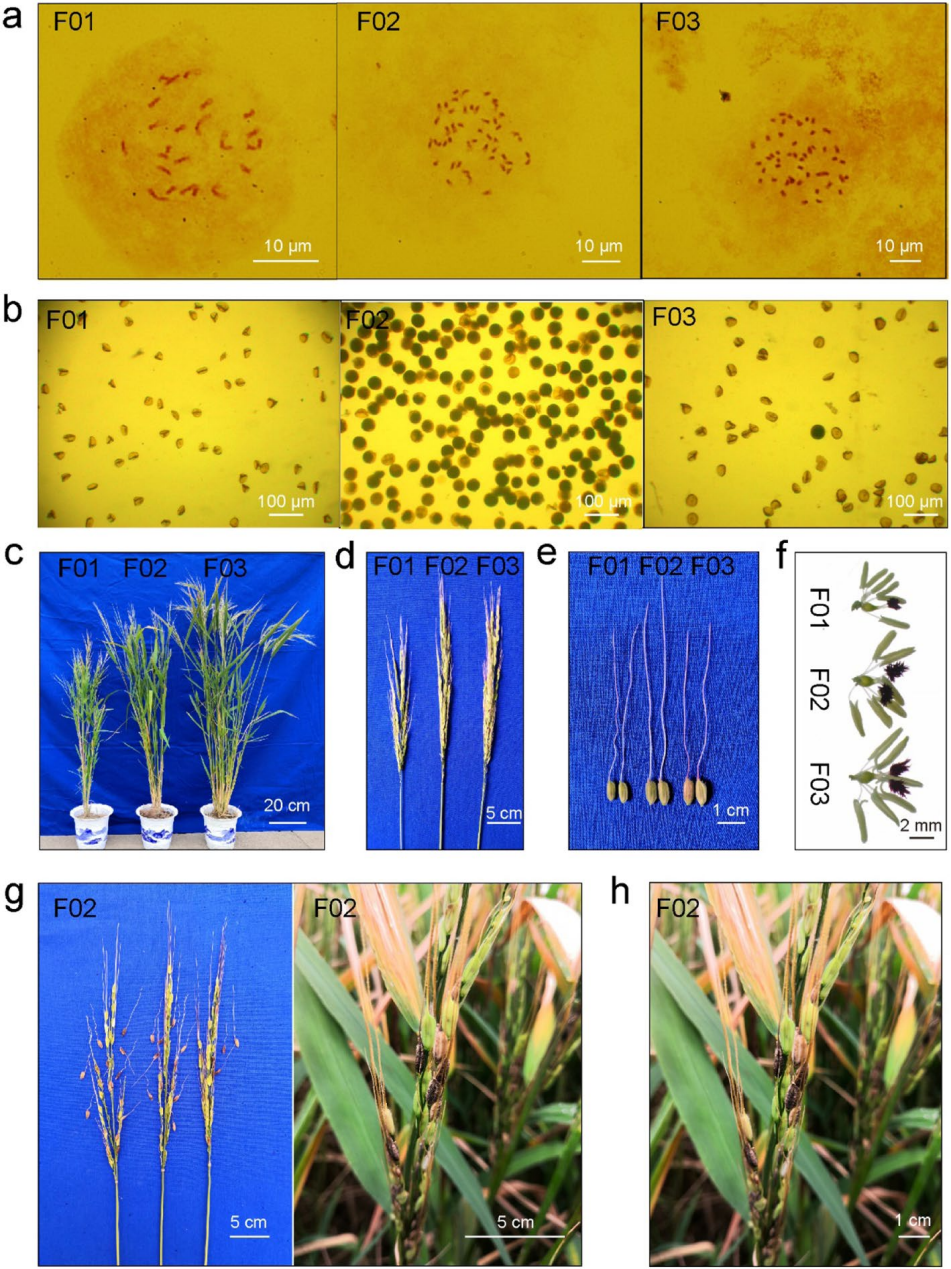
General characteristics of different ploidy and genome compositions hybrids. **a** Chromosomes. **b** Pollens. **c** Plants. **d** Panicles. **e** Grains. **f** Pistil and stamen. **g** Shattering of F02. **h** Black grain of F02

As shown in Fig. 1a, the number of chromosomes in the different hybrid cells was 24 in F01 (AB), 48 in F02 (AABB) and 48 in F03 (AAAB) according to the chromosomes found in the root tips. With the multiplication of chromosomes, there were obvious differences in the external morphology of F01 and F02 (Fig. 1b). The allotetraploid hybrid plants (F02) were taller and had sturdier stems, larger, thicker and darker colored leaves and larger panicles, grains and floral organs compared with the hybrid F01 (Fig. 1c ~ h). In addition, the amphiploid plants exhibited stronger growth, showing the obvious “giant” effect of polyploid organs (Table 1). In terms of seed setting, the allotetraploid hybrid F02 (AABB) had a seed setting rate of 53.16%, while the diploid hybrid F01 could not produce seed at all. The hybrids with the same ploidy (F02 and F03) also showed significant morphological differences due to the different genome compositions (Fig. 1). Compared with F02, the allotetraploid hybrid F03 grew taller, had larger panicles and floral organs and contained more grains per panicle. The overall phenotype of F03 (AAAB) was more similar to that of cultivated rice due to its increased repetition of the A genome. In terms of fertility, F03 did not set seed after selfing, although immature embryos were occasionally observed. The common features of F01, F02 and F03 were that they all had purple stigmata, red awns, black chaffs and displayed easy shattering.

**Table 1:** Comparison of morphology characteristics between different ploidy and genome compositions hybrids

Materials	F01 (AB)	F02 (AABB)	F03 (AAAB)
Plant height (cm)	93.51 ± 4.02	103.00 ± 1.25	109.20 ± 2.29
Panicle no. per plant	24.16 ± 0.37	17.57 ± 1.05	19.86 ± 1.45
Panicle length (cm)	20.01 ± 1.13	25.88 ± 1.97	31.57 ± 1.17
Grain length/width (cm)	0.70/0.29	0.90/0.30	0.90/0.35
Awn length (cm)	6.10 ± 0.30	6.90 ± 0.23	7.51 ± 0.41
Total grain no. per panicle	170.00 ± 6.32	116.35 ± 5.64	187.29 ± 7.73
Seed setting rate (%)	0	53.16 ± 2.14	0
Flag leaf length (cm)	15.6 ± 1.37	19.4 ± 0.68	20.1 ± 0.39
Flag leaf width (cm)	2.3 ± 0.25	2.5 ± 0.24	2.1 ± 0.12
Shattering trait	Shattering	Shattering	Shattering
Awn color	Red	Red	Red
stigma color	Purple	Purple	Purple
Seed color	Black	Black	Black

The mature and normal pollen grains in the allotetraploid hybrid F02 were full, round, dark and 41.44 μm in mean diameter. The pollen grains of F01 and F03 could not be stained, and typical abortive pollen was the main type, showing characteristics of shrinkage and shriveling. Therefore, the diameters of the pollen grains were smaller, at 21.08 μm and 26.21 μm, respectively (Table 2). There were also obvious differences in pollen fertility among different ploidy hybrids (Table 2, Fig. 1). There were almost no stained pollens in F01. A few stained pollens appeared in F03, and the highest staining rate was 4.35%. The pollen staining rate for F02 was the highest, ranging from 40.11% to 80.26%, with an average of 62.89%. The pollen staining rate can directly reflect the fertility characteristics of the material. According to the results of pollen staining, F02 had the highest level of fertility, while F01 and F03 had poor fertility, which was consistent with the results of the investigation on seed setting rates.

**Table 2:** Comparison of pollen diameter and fertility between different ploidy and genome compositions hybrids

Materials	Pollen diameter			Pollen staining rate		
	**Min (μm)**	**Max (μm)**	**Average (μm)**	**Min %**	**Max %**	**Average %**
F01 (AB)	16.12	30.22	21.08 ± 3.91	0.00	0.00	0.00
F02 (AABB)	29.42	49.01	41.44 ± 6.67	40.11	80.26	62.89 ± 16.97
F03 (AAAB)	19.75	38.08	26.21 ± 5.18	0.00	4.35	0.93 ± 1.57

### Full-length transcriptome sequencing analysis with different ploidy levels

In this study we applied the Iso-seq approach to a transcriptomic analysis of rice hybrids with different ploidy and genome compositions (Fig. S1), which had polyploid genomes of greater complexity than those of Asian cultivated and wild rice lines [28]. To develop a comprehensive catalog of transcript isoforms, high-quality RNA was extracted from five tissues of the hybrids. These tissues were sampled at different developmental stages and then pooled to construct Iso-seq size-fractionated libraries (1–6 kb). After quality control (Figs. S1 and S2), the PacBio RS II platform generated circular consensus (CCS) reads of 278,855 (F01), 290,833 (F02) and 293,637 (F03), including full-length non-chimeric reads of 244,819 (87.78%), 257,816 (88.65%) and 258,052 (87.88%) based on the presence of 5' primers, 3' primers and poly (A) tails (Table 3). The full-length non-chimeric reads were further clustered to obtain consensus isoforms, and the consensus isoforms in each cluster were corrected to obtain high-quality isoforms of 31,153 (F01), 30,603 (F02) and 28,840 (F03), respectively. By removing redundancy, non-redundant consensus isoforms of 11,223 (F01), 12,722 (F02) and 13,472 (F03) were obtained, covering 8336, 8767 and 9140 gene loci, respectively. At the same time, 270 (F01), 206 (F02) and 259 (F03) new gene loci and 2746, 4044 and 4113 new isoforms were found. These results showed that the non-redundant isoforms, gene loci, lengths of full-length transcripts and new isoforms in polyploid rice were higher than those in the diploid hybrid F01. The 1700 bp length of the polyploid isoforms was much longer than that of the Ensembl Plants (mean 1190 bp). These Iso-seq full-length isoforms produced directly from sequencing without assembly are valuable resources for optimizing gene models.

**Table 3:** Statistics of the SMRT sequencing data with different ploidy and genome compositions hybrids

Items	F01 (AB)	F02 (AABB)	F03 (AAAB)
Number of circular consensus reads	278,855	290,833	293,637
Non-full-length transcripts	244,819	257,816	258,052
Filtered short reads	174	81	65
Number of consensus isoforms	31,409	30,778	28,933
Average consensus isoforms read length	1127	1710	1760
Nonredundant isoforms	11,223	12,722	13,472
Gene loci	8336	8767	9140
New gene loci	270	206	259
New isoforms	2746	4044	4113

### Splice junctions and AS modes with different ploidy levels

**Fig. 2:**
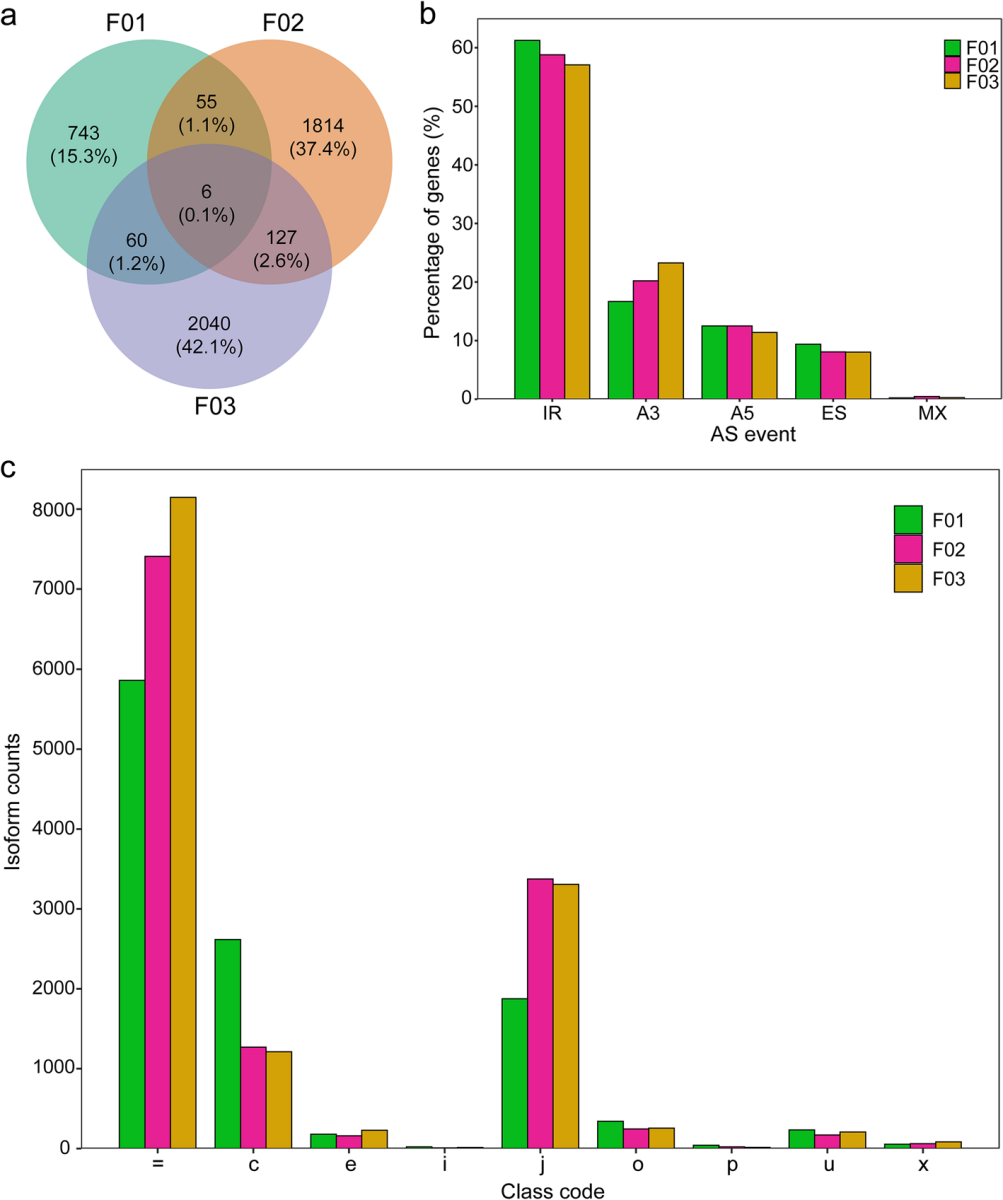
Identification of Alternative splicing (AS) events of different ploidy and genome compositions hybrids. **a** Venn diagram of isoforms between different ploidy hybrids. **b** Distribution of isoforms that produce one or more splice isoforms from Iso-Seq data. **c** Information of isoforms with class codes

Isoform sequencing technology yields long reads without the aid of assembly and provides superior evidence for identifying AS variants. Based on obtaining high-quality full-length isoforms, we systematically analyzed AS events. Five major AS events were identified, including IR, A5, A3, ES and MX events, by customizing a user-friendly program. A total of 480, 1104 and 1195 AS events formed 864, 2002 and 2233 alternative splice variants from the three samples, respectively, with few proportions of shared splice variants (Fig. 2a). Figure 2b indicates that the main IR events accounted for 60%, the ratio of A3 in polyploids was significantly higher than that in the diploid, and the ratio in F03 was higher than that in F02. The ratios of ES and IR events were significantly lower than those in the diploid, and the ratios in F03 (AAAB) were lower than those in F02 (AABB). Therefore, AS events occurred twice as often in the polyploids than in the diploid rice. There was no significant difference between F02 (AABB) and F03 (AAAB).

Here, the transcripts with a class code of “ = ” were defined as “known transcripts”, whereas all others (such as “c”, “i”, “p”, “j”, “u”, “e”, “x” and “o”) were defined as “unannotated isoforms” using Cuffcompare. Figure 2c shows 2746, 4043 and 4113 unannotated isoforms, respectively. For example, a gene *LOC_Os01g31360* that is annotated to possess five transcripts was found to generate 13 splice isoforms from the Iso-seq data of F02 (PB.415 splice isoforms, Tables S1 and S2). A gene *LOC_Os08g06110* that is annotated to possess five transcripts was found to generate 15 splice isoforms from the Iso-seq data of F03 (Table S2). It was therefore suggested that AS events led to complex transcriptional regulation in polyploid rice. The genes of *LOC_Os01g31360* and *LOC_Os08g06110* were used to validate the accuracy of AS events using reverse transcription polymerase chain reaction (RT)-PCR. Isoforms of each gene were aligned in order to design primers that could amplify all predicted transcripts at the same time (Table S4). We observed sequence consistency between cloned fragments and predicted sequences based on Iso-Seq data (Fig. S4).

### Alternative polyadenylation analysis

**Fig. 3:**
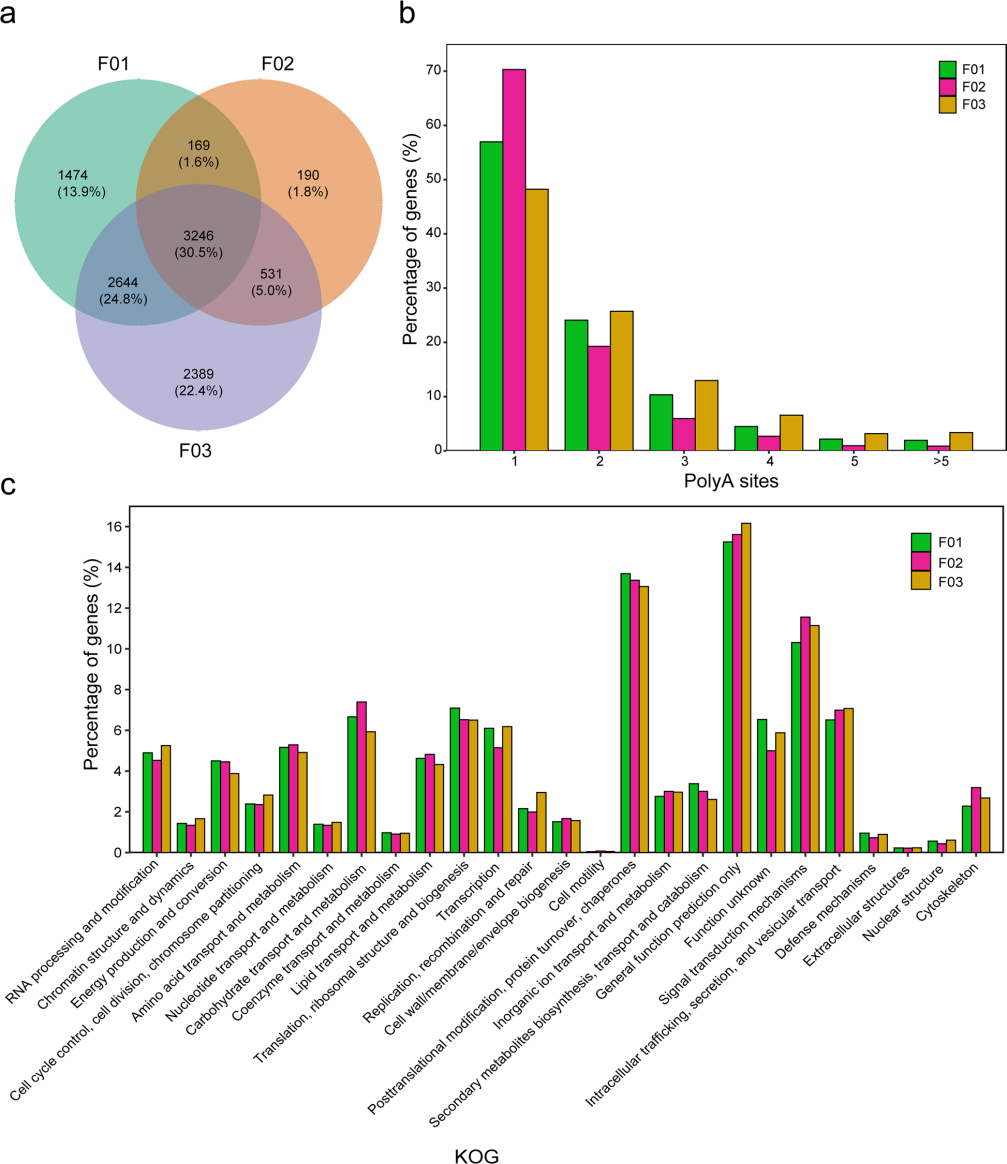
Alternative polyadenylation (APA) events analysis of different ploidy and genome compositions hybrids. **a** Venn diagram depiction common and unique isoforms of APA of different ploidy and genome compositions hybrids. **b** Percentage of isoforms with different numbers of poly (A) sites. **c** KOG functional classification of APA isoforms

Many studies have shown that APA events increase transcriptome complexity and can regulate gene expression [25]_._ APA analysis is subject to certain limitations using conventional RNA-seq short read sequences, and the effect of APA on the complexity in lines of different ploidy are still unknown in rice. The investigation of 3' ends of transcripts using Iso-seq allowed us accurately to identify differential polyadenylation sites in rice (Fig. 3). In our study, in total 13,468, 6126 and 18,038 APA sites were determined in the hybrid rices F01 F02, F03, respectively. On average, 1.79, 1.48 and 2.05 poly (A) sites per gene were found in the hybrid rices F01 F02, F03, respectively (Fig. 3b). Additionally, 7532, 4135 and 8810 genes contained at least one APA site and 146, 36 and 297 genes had at least five poly(A) sites in the hybrid rices F01 F02, F03, respectively. These results suggested that that APA is a common phenomenon in rice.

Figure 3a shows the 3246 genes shared by APA sites in the three samples, which account for 30.5%. There were more unique genes in F03 than F01. The proportion of isoforms in the KOG category was different for different ploidy levels (Fig. 3c). The ratio of enriched genes in carbohydrate transport and metabolism, lipid transport and metabolism, signal transduction mechanisms and cytoskeleton were the highest in F02 and lowest in F01. However, the ratio of enriched genes in signal transduction mechanisms and intracellular trafficking, secretion and vesicular transport in allotetraploid hybrid was greater than that in the diploid hybrid F01. Polyploidy and hybridization with variable levels of polyadenylation have certain effects on plant growth, transcription and repair. The results indicated that the polyadenylation of these genes has a certain regulatory effect, affecting biological processes such as plant growth, development, stress responses and other biological processes, while also affecting the diversity and complexity of polyploid genetic traits.

### Functional annotation with different ploidy levels

**Fig. 4:**
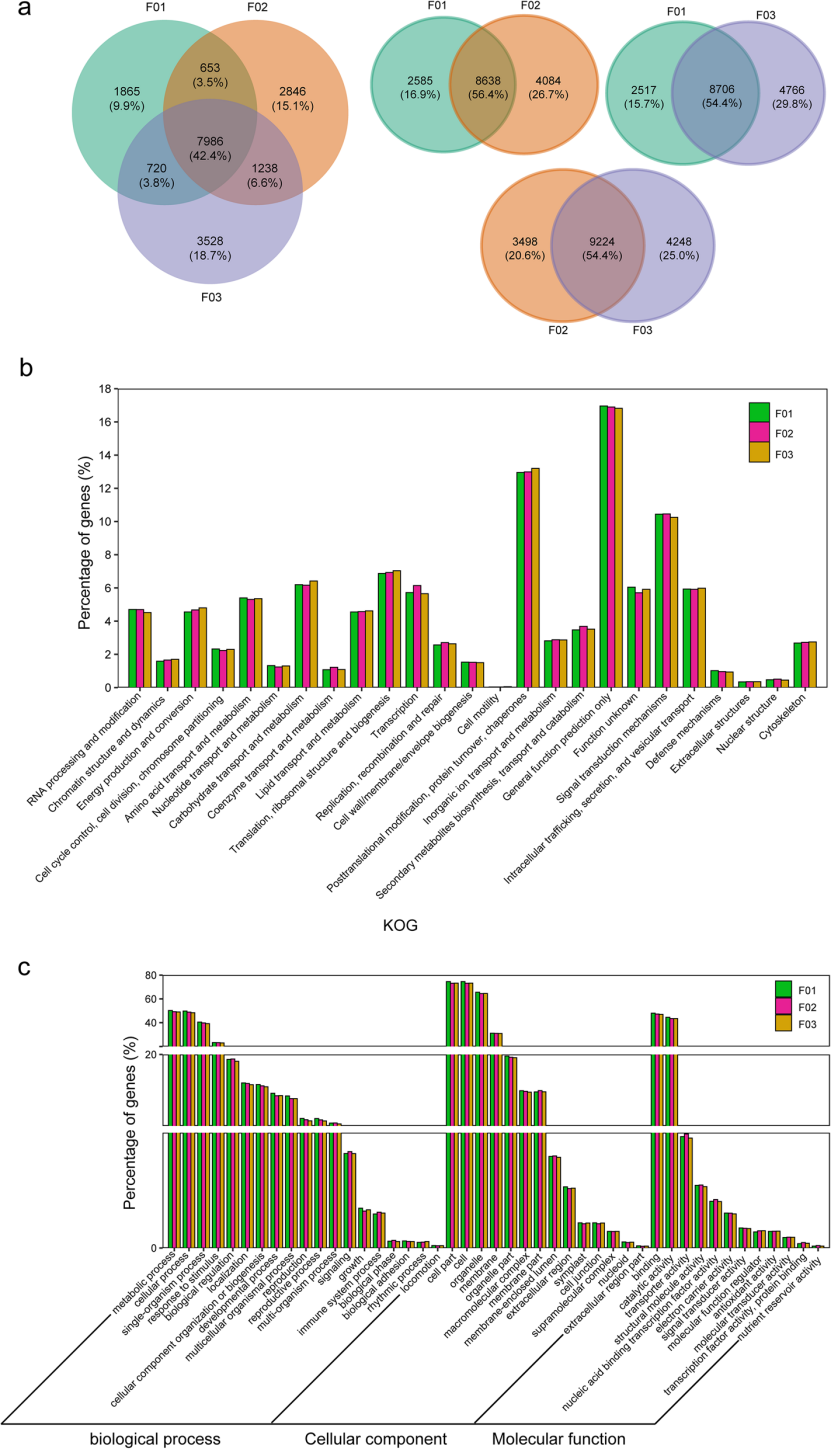
Functional annotation of isoforms with different ploidy and genome compositions hybrids. **a** Venn diagram of the non-redundant isoforms with different ploidy and genome compositions hybrids. **b** KOG functional classification of isoforms. **c** Comparison of Gene Ontology (GO) classifications of isoforms

The non-redundant isoforms of different ploidy levels were 11,223, 12,722 and 13,472 (Fig. 4a), respectively. The common isoforms were 7986 (42.4%) within the three samples, 8638 (56.4%) for F01 and F02, 8706 (53.5%) for F01 and F03, and 9224 (54.4%) for F02 and F03. More than half of the isoforms were mutually shared, and about 45% of the isoforms were mutually exclusive. The unique isoforms increased between the change from F01 to F02, indicating that polyploidization involves complex transcriptional regulation. Non-redundant transcripts were annotated with COG, KOG, GO and KEGG functions, and the annotation rates were 79.5%, 75.5% and 75.10%, respectively, indicating that full-length transcriptome sequencing plays a very powerful role in the study of post-transcriptional modification, new gene discovery and genome annotation.

The number of isoforms for different ploidy levels was basically the same under normal growth conditions through KOG functional classification (Fig. S3), and the main enrichment categories were nearly consistent with the different ploidy levels. From F01 to F03, the number of isoforms in the KOG categories increased. The ratio of isoforms in the KOG categories related to energy production and conversion, transcription and replication, recombination and repair significantly increased from F02 to F01 (Fig. 4b). Meanwhile, the ratio of isoforms in the KOG categories related to energy production and conversion, carbohydrate transport and metabolism, and translation, ribosomal structure and biogenesis in F03 was higher than that in F02, and the ratio of isoforms in the KOG categories related to transcription in F03 was lower than that in F02, indicating that hybridization offers advantages to plant growth. In the COG categories, the ratio of isoforms related to carbohydrate transport and metabolism, coenzyme transport and metabolism, lipid transport and metabolism and translation, ribosomal structure and biogenesis significantly increased from F02 to F01. This may be the reason why the polyploid rice grew tall and sturdy. The results from the COG analysis suggested the function of translation, ribosomal structure and biogenesis was more complex for allotetraploid rice lines. Figure 4c and Fig. S4 shows that the number of isoforms in the allotetraploid hybrid was higher than that in diploid hybrid F01, and that in allotetraploid hybrid F03 was greater than in F02. However, the proportion of most GO terms did not change significantly (Fig. S5). Only the ratios of transporter activity, nucleic acid binding transcription factor activity and molecular function regulators in allotetraploid rice were higher than in the diploid hybrid F01. This suggested that APA and AS events may contribute to the complexity of polyploidy in translation, nucleic acid binding transcription factor activity and molecular function regulation.

**Fig. 5:**
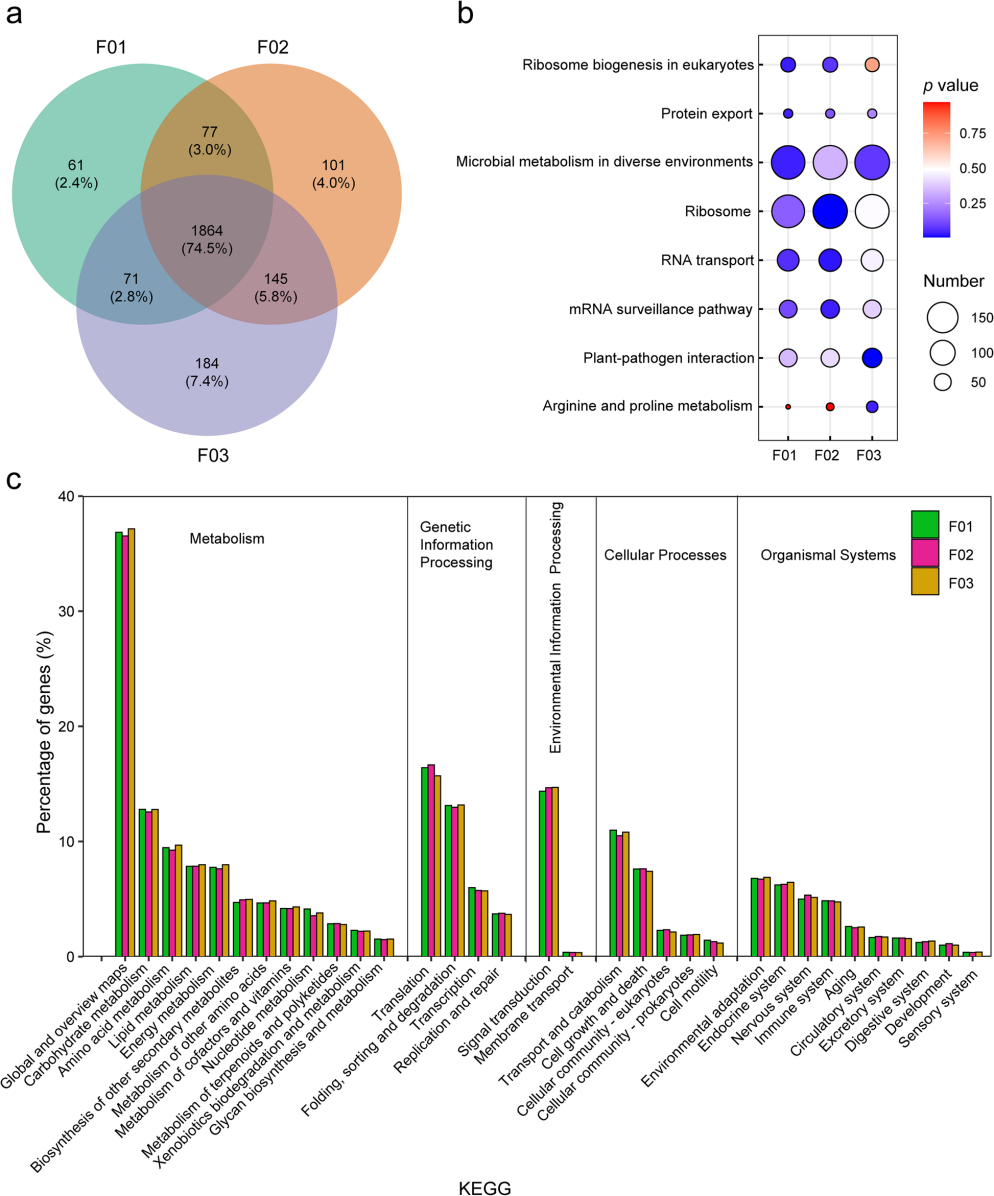
KEGG enrichment analysis of isoforms with different ploidy and genome compositions hybrids. **a** Venn diagram of the isoforms of KEGG enrichment. **b** Statistics of pathway enrichment (*P* < 0.05), the number of isoforms is distinguished by the size of the circle and the circle from blue to red represents the *P*-value from large to small. **c** Comparison of KEGG classifications of isoforms

Figure 5 shows that the metabolic pathways and isoforms of rice with different ploidy were mostly the same. Among the lines of different ploidy, 74.5% of isoforms were common, whereas 25.5% were specific. Although the specific isoforms of KEGG enrichment in allotetraploid hybrid were greater in number than those of the diploid hybrid F01, they accounted for a smaller proportion (Fig. 5a). In the KEGG enrichment terms, the change in the number of isoforms was the same as that for the KOG and GO terms; the number of isoforms for each term increased gradually between F01 and F03 (Fig. S3). These isoforms belonged mainly to the following KEGG pathways (Fig. 5c): biosynthesis of secondary metabolites, biosynthesis of antibiotics, carbon metabolism, biosynthesis of amino acids and protein processing in endoplasmic reticulum and spliceosome. The percentage of isoforms participating in carbohydrate metabolism, amino acid metabolism and energy metabolism in F02 was lower than that in F01, but the percentage of isoforms participating in translation and signal transduction in F02 was higher than that in F01. In F03, the percentage of isoforms participating in most of pathways (especially carbohydrate metabolism, amino acid metabolism and energy metabolism) was greater than that in F02. Three KEGG pathways including ribosome biogenesis in eukaryotes, protein export and microbial metabolism in diverse environments were significantly enriched in F01 (Fig. 5b). Two KEGG pathways including RNA transport and mRNA surveillance were significantly enriched in F02, and two KEGG pathways including plant-pathogen interaction and arginine and proline metabolism were significantly enriched in F03. These findings confirmed that AS events and APA play certain regulatory roles.

### New isoforms with different ploidy levels

**Fig. 6:**
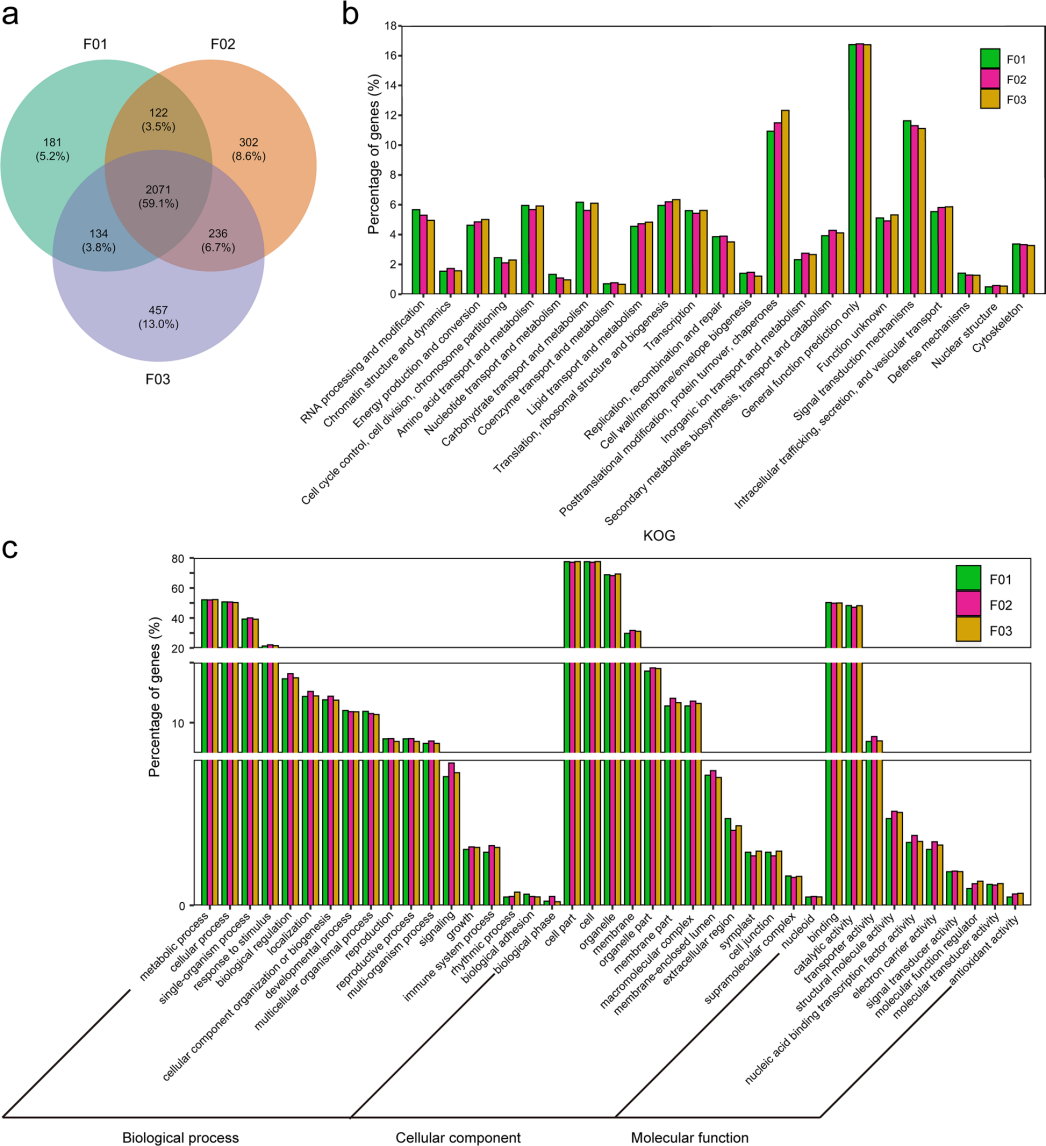
New isoforms analysis with different ploidy and genome compositions hybrids. a Venn diagram of the new isoforms. b KOG functional classification of new isoforms. c Comparison of Gene Ontology (GO) classifications of new isoforms

In total, 2746, 4044 and 4113 new isoforms were discovered using Iso-seq data. There was a higher number of these in the polyploids (F02 and F03) than the diploids, but there was no significant difference between F02 and F03 (Fig. 6a). Over half [2071 (59.1%)] of the new isoforms were common. This indicated that more new isoforms can be found through SMRT sequencing, and the number of new isoforms was less affected without the addition of new genes.

The new isoforms were KOG-annotated (Fig. 6b). The percentage of new isoforms participating in energy production and conversion, lipid transport and metabolism, translation, ribosomal structure and biogenesis, posttranslational modification, protein turnover, chaperones, secondary metabolites biosynthesis, transport and catabolism and intracellular trafficking, secretion, and vesicular transport in polyploids was higher than that in the diploid hybrid, and it gradually increased from F01 to F03. In GO terms of the new isoforms (Fig. 6c), the number of new isoforms also gradually increased from F01 to F03. In biological processes, the new isoforms belonging to the following GO terms: response to stimulus, biological regulation, signaling, growth, immune system process in polyploids were greater in number than those of the diploid hybrid, and those in F02 were greater than those in F03. In cellular components, the percentage of new isoforms belonging to the following GO terms: membrane part, macromolecular complex and membrane-enclosed lumen in the polyploids was higher than that in the diploid hybrid. In molecular function, the percentage of new isoforms belonging to most of the GO terms in the polyploids was higher than that in the diploid hybrid. These results indicated that polyploidy was associated with obvious regulatory complexity and high levels of growth response to external stimuli from the new isoforms.

### Expression analysis of genes associated with heading period and pollen development

**Fig. 7:**
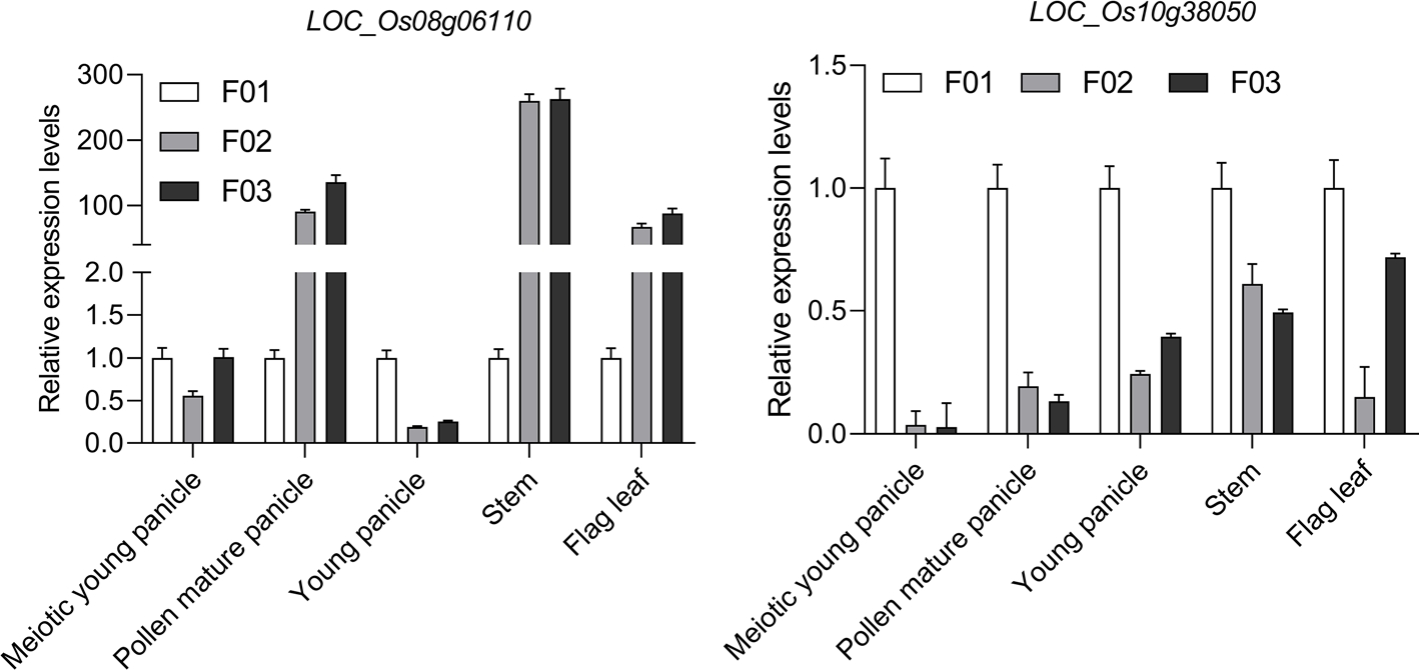
Validation of expression patterns of *LOC_Os08g06110* and *LOC_Os10g38050* by qRT-PCR. The relative expression levels were calculated

The rice heading period could be regulated by gene *LOC_Os08g06110* through sensing changes in the length of the day (photoperiod). In this study, Fig. 7 showed the relative expression levels of *LOC_Os08g06110* of F02 and F03 in the pollen mature panicle, stem and flag leaf were higher than that of F01. This is consistent with the characteristic that F02 and F03 have a delayed heading date than F01 under long-day conditions (≥ 12 h). The pollen development could be regulated by gene *LOC_Os10g38050*, resulting in a small amount of pollen. The relative expression levels of *LOC_Os10g38050* of F01 were highest, resulting the small amount of pollen and infertility. On the contrary, the pollen number of F02 and F03 was high due to low expression levels.

## Discussion

Hybridization and polyploidy are effective ways to increase plant biomass, yield and resistance to biotic and abiotic stresses [13, 29, 30]. Using Asian cultivated rice (*O. sativa*) and wild rice (*O. punctata*) as the basic materials, a series of resources created by hybridization can be produced that retain the excellent characteristics of the parents. Currently, our understanding of the rice transcriptome is based mainly on SGS technologies (including resequencing) [31, 32]. Therefore, the transcriptome of rice has not been fully identified due to the lack of full-length cDNA. In this study, we conducted a comprehensive investigation of the transcriptome of rice with different ploidy using PacBio third-generation sequencing. In total, 11,223, 12,722 and 13,472 non-redundant isoforms were obtained, covering 8336, 8767 and 9140 gene loci, respectively. In addition, 2746, 4044 and 4113 new isoforms and 270, 206 and 259 new gene loci were found. New isoforms may be involved in posttranslational modification, protein turnover, chaperone proteins, signal transduction mechanisms and response to stimuli. These new findings provide important information for improving the genome annotation of polyploid rice and comprehensively describing the rice transcriptome.

### A new pathway for creating novel crops

Under the guidance of the strategy ‘breeding super rice by using double advantages of distant hybridization and polyploidization’ [10], a number of allopolyploid rice lines including the materials used in this study were developed. These allopolyploids greatly enriched the germplasm resources of rice breeding, and provided materials for making full use of the elite genes of wild rice and the study of evolution of *Oryza* species. The results showed that the allotetraploid hybrid AABB (F02) not only overcomes the sterility of the distant hybrid AB (F01) but also displays strong heterosis. Nearly 20 years of sexual reproduction proves that it can be inherited stably in our laboratory. The breeding practice of AABB shows the feasibility of the pathway ‘breeding super rice by using double advantages of distant hybridization and polyploidization’. This demonstrates a new pathway for creating novel crops in the future. Crop polyploidization may play an important role in next-generation crop improvement aimed at facing food security challenges [7].

### Complexity of AS in allotetraploid hybrids

Hybridization and polyploidization events have frequently occurred throughout plant evolutionary history, and they have played an important role in the evolution of species and the formation of new species. AS is a unique posttranscriptional modification in eukaryotes, by which a gene produces multiple transcriptional translations, creating complexity in the eukaryotic transcriptome. AS in plants can be regulated by many mechanisms initiated at different developmental stages and by environmental signals. These events play an important role in abiotic stress, biotic stress, hormone regulation, development, flowering time and the biological clock response. Therefore, AS may also be an indispensable part of the mechanism of heterosis. In general, the type of AS in plants is mainly IR [26, 33, 34]. In this study, IR events accounted for about 60% of AS incidents, which is similar to that found in other plants such as *Zea mays* and cotton [25, 35]. Wang et al. confirmed that AS greatly increases the complexity of gene transcription in polyploid cotton [25]. The number of AS events in the polyploids (F02, F03) increased compared with those in the diploid hybrid (F01). This indicated that AS events are not very conservative among different rice samples. The number of AS events occurring during polyploidization was higher than that during hybridization. Therefore, hybridization and polyploidization impacted AS.

### Complexity of APA in allotetraploid hybrids

In eukaryotes, polyadenylation affects the localization, stability, translation efficiency and function of RNA and is an important method of posttranscriptional modification and regulation, which in turn regulates gene expression through a variety of regulatory mechanisms [36, 37], increasing the complexity of the transcriptome. For example, polyadenylation of mRNA plays an important role in the regulation of plant growth and development, especially in the flowering process [38]. The PacBio Iso-seq Platform is very suitable for the accurate reconstruction of full-length splice variants [24, 33]. Abdel-Ghany et al. found the transcripts of sorghum had widespread APA events (about 50% of expressed genes with multiple poly(A) sites) [33]. In this study, we report for the first time the polyadenylation events of the whole genome map in allotetraploid hybrid rice using SMRT sequencing (Table S3). The number of poly(A) sites found in the allotetraploid hybrid F02 was lower than in the polyploid F03, and the genes at these sites showed significant differences in functional annotations, mainly during RNA processing and modification, transcription, replication, recombination and repair, carbohydrate transport and metabolism, lipid transport and metabolism, signal transduction mechanisms and cytoskeleton functions. The results indicated that hybridization also significantly affected the poly(A) sites, and the poly(A) sites do not necessarily increase with increasing chromosome multiplication.

### Differences in transcriptional function of rice with different ploidy

Tetraploids generally show vegetative growth superiority, larger organs, higher biological yield, more secondary metabolites and enhanced resistance than diploids [39, 40], which is already important in plant breeding. Therefore, the mechanisms underlying the polyploid characteristics are being revealed gradually [41]. Recently, the dominant mechanism of tetraploid rice has been revealed [42–44]. The mechanism of the advantage of long panicles in neo-tetraploid rice (Huaduo 8) had revealed using transcriptomics [43]. And, the genes associated with fertility and yield had been found by comparing genomes in neo-tetraploid rice [44]. Therefore, the molecular genetic mechanism involved in heterosis of tetraploid rice is very complex [31]. For example, genes associated with energy metabolism and transport enriched in differentially expressed genes between the hybrid and its parents rather than in differentially expressed genes between the parental lines, and differentially expressed genes between the hybrid and its parents were significantly enriched in carbohydrate metabolism and plant hormone signal transduction [45]. In this study, polyploidy not only increased the number of genes involved in many pathways but also increased the proportion of genes involved in energy and carbohydrate use. This may be the reason why leaves of polyploids become larger and thicker, while the plants grow taller and stronger. In addition, isoforms in polyploids were enriched in RNA transport and the mRNA surveillance pathway, while the AS and APA events in allopolyploid rice played a role in regulation.

## Conclusion

In this study, we comprehensively investigated the transcriptome of the distant hybrids with different ploidy and genome compositions using PacBio third-generation sequencing. Allotetraploid hybrid (AABB) had a good phenotype in plant growth and high rate of seed setting, and could be used as a breeding resource. Furthermore, genetic differences and dominance were revealed by the full-length transcriptome. Allopolyploidy not only increased the number of genes involved in many pathways but also increased the proportion of isoforms involved in energy and carbohydrate use. Alternative transcripts were existed in post-translational modification, protein turnover, chaperones, signal transduction mechanisms and responses to stimuli. These new findings will provide important information for improving genome reannotation of polyploid rice and strengthen our understanding of the rice transcriptome.

## Materials and methods

### Plant materials

Asian cultivated rice *O. sativa* (AA, 2n = 2x = 24) subsp. *japonica* was stored at the Polyploid Genetics Lab of Hubei University, Wuhan, China. Wild rice *O. punctata*, (BB, 2n = 2x = 24, IRRI Number: 105980) was kindly provided by the International Rice Research Institute (IRRI), Manila, Philippines. The diploid interspecific hybrid F01 (AB, 2n = 2x = 24) was obtained by crossing *O. sativa* × *O. punctata*. F02, an allotetraploid hybrid (2n = 4x = 48, AABB), was obtained via chromosome doubling of F01(AB), and F03, an allotetraploid hybrid (AAAB) was obtained by crossing tetraploid *O. sativa* (AAAA) × F02 (AABB).

### Cytogenetic analysis

Chromosome root tip squashes were made according to the method described by Wang et al. [13] with minor modifications. Root tips were excised from the plants and pretreated with 2 mM 8-hydroxyquinoline for 2 h at room temperature and fixed in fresh Carnoy’s fluid [methanol/acetic acid, 3:1 (v/v)] overnight. Then the root tips were rinsed in 75 mM KCl for 30 min at room temperature, digested in an enzyme mixture containing 2% cellulase and 2% pectinase at 28 °C for 4 h, washed three times in distilled water and incubated in distilled water at room temperature for 20 min. These root tips were then placed on pre-cooled slides and squashed in the presence of a fixative. The slides were heated over an alcohol flame to dry the fixative, stained with carbolfuchsin, washed with a fresh stream of water and then dried at room temperature. The chromosomes were observed under an Olympus BX51 microscope (Olympus Corporation, Japan) and photographed.

### Morphological and agronomic characteristics

Morphological and agronomic characteristics, such as plant height (*n* = 5), flag leaf length and width (*n* = 5), panicle number per plant (*n* = 5), panicle length (*n* = 25), grain length and width (*n* = 25), awn length and color (*n* = 25), total grain number per panicle (*n* = 25), filled grain number per panicle (*n* = 25), seed setting rate (filled grain number/total grain number), stigma color, grain color and shattering traits were measured.

Mature pollen grains were stained with 1% I_2_-KI solution and observed under an Olympus BX51 microscope to analyze their fertility. Three slides were made for each observation and pollens were randomly counted in 10 micro-optical fields on each slide. The diameters of 30 pollen grains, chosen at random, were measured with a micrometer per slide. The ratio of fertile pollen (%) = (the no. of stained pollen grains/the no. of total pollen grains) × 100.

### Extraction of RNA

Total RNA was extracted by grinding tissue in TRIZOL reagent. To determine the RNA quality, samples were assessed using a NanoDrop microspectrophotometer (Thermo Fisher Scientific) and an Agilent 2100 Bioanalyzer (Agilent Technologies). The total RNA from five tissues including the flag leaf, stems, young panicles, meiotic young panicles and pollen mature panicles was pooled together in equal amounts.

### Library preparation and isoform sequencing

The mixed RNA samples were reverse transcribed into cDNA using the SMARTer® PCR cDNA synthesis kit and optimized to prepare high-quality and full-length (FL) cDNAs. Subsequently, size fractionation and selection (1–6 kb) were conducted using the BluePippin Size-Selection System (Sage Science, Beverly, MA). A Single Molecule, Real-Time (SMRT) bell library was constructed with the PacBio DNA Template Prep Kit 2.0. The library was subsequently sequenced on the PacBio RS II platform with P6C4 polymerase enzyme and chemistry.

### Quality filtering and error correction

Raw reads were processed into error-corrected reads of insert (ROIs) using the isoform sequencing (Iso-seq) pipeline with min Full Pass = 0 and min Predicted Accuracy = 0.80. Next, full-length, non-chimeric transcripts were determined by searching for the polyA tail signal and the 5' and 3' cDNA primers in the ROIs. Iterative Clustering for Error Correction (ICE) was used to obtain consensus isoforms, and FL consensus sequences were polished using Quiver. High quality FL transcripts were classified with the criteria of post-correction accuracy above 99%.

FL consensus sequences were mapped to the reference genome (*Oryza sativa* L. spp. *japonica *var. Nipponbare *MSU_v7.0* ftp://ftp.plantbiology.msu.edu/pub/data/Eukaryotic_Projects/o_sativa/annotation_dbs/pseudomolecules/version_7.0/all.dir/) using Genomic Mapping and Alignment Program. Mapped reads were further collapsed using the pbtranscript-ToFU package with min-coverage = 85% and min-identity = 90%. The 5' difference was not considered when collapsing redundant transcripts.

### Characterization of AS events

The determination of AS events was carried out using the Astalavista tool [46] with default parameters. The .gtf file from each assembly was used as the input. The output provided for all the AS events from the whole transcriptome data was further analyzed manually.

We divided the AS events into five different types according to the structure of the exon [47]. Introns fully subsumed by an exon were labelled as retained (intron retention, IR). Overlapping exons that differed at their 5' or 3' splice junctions were considered alternative 5' or 3' splicing events (alternative 5' splice site, A5; alternative 3' splice site, A3), respectively. Exons absent in other isoforms were considered exon skipping events (exon skipping, ES). The constitutive exon cannot coexist in the same transcript as mutually exclusive exons (MX).

We used the Cuffcompare utility in the Tuxedo suite to categorize each long-read transcript with respect to its most closely matching reference transcript [48]. The Cuffcompare class codes underlying long-read transcript classification were: “u” for “Potentially novel gene”; the set of “e” and “j” for “Potentially novel isoform or inaccurate reference”; “ = ” for “Exact match to annotation”; “c” for “Sequential subset of exons contained within annotation”; and the set of “I”, “o”, “p”, “r”, “s” and “x” for “Other transcripts”. The code “e” (88 transcripts), defined as “Single exon transfrag overlapping a reference exon and at least 10 bp of a reference intron” was added to the “Potentially novel isoform or inaccurate reference” category.

### Alternative polyadenylation analysis from PacBio data

Sites of APA and unannotated genes were identified using Transcriptome Analysis Pipeline for Isoform Sequencing (TAPIS) software [33] and PRAPI software [49]. Only consistent results detected from both were retained for further analysis.

### Functional annotation

Corrected isoforms were searched against NCBI non-redundant (NR), NCBI nucleotide sequence (NT), Swiss-Prot (a manually annotated and reviewed protein sequence database), Cluster of Orthologous Groups (KOG/COG) [50] and Kyoto Encyclopedia of Genes and Genomes (KEGG) [51] databases with BLAST software. Gene Ontology (GO) annotations were determined based on the best BLASTX hit from the NR database using the Blast2GO software [52]. KEGG pathway analyses were performed using KOBAS 3.0 software (http://kobas.cbi.pku.edu.cn/index.php) [53, 54], and HMMER software was used to search the Pfam database [55].

### Validation by RT-PCR

To verify the AS events detected above, we designed primers for the AS regions of these (Table S4). Total RNA was isolated from the same samples as described above for Iso-Seq using TRIZOL reagent (Invitrogen, https://www.invitrogen.com). Complementary DNA was synthesized from 1 μg total RNA in a 20 μl solution using the MonScript™ RTIII Super Mix with dsDNase (Two-Step) (MR05201, Monad, http://www.monadbiotech.com).

Primer3Plus webpage (http://www.primer3plus.com/cgi-bin/dev/primer3plus.cgi) was applied to design the primers for qPCR. The PCR was used to identify the specificity of the primers. Gimmico A4 Fidelity PCR Mix (1.1 ×) (GM1104, Gimmico, https://www.gimmico.cn) was used for PCR amplification. The PCR products were visualized in agarose gel stained with SuperRed/GelRed (BS354B, Biosharp, https://www.biosharp.cn). MonAmp™ SYBR® Green qPCR Mix (MQ10201S, Monad, http://www.monadbiotech.com) was used for qPCR amplification. The qPCR was carried out using cDNA, a gene-specific forward primer and a gene-specific reverse primer. The β-actin was amplified as an endogenous control.

Primer3Plus webpage (http://www.primer3plus.com/cgi-bin/dev/primer3plus.cgi) was applied to design the primers for the gene cloning. I-5™ 2 × High-Fidelity Master Mix (TP001, Tsingke, https://tsingke.com.cn/products/details/6) was used for PCR amplification. The PCR was carried out using cDNA, a forward primer and a reverse primer. PCR amplification was monitored on 1.5% agarose gel and the PCR products were visualized in agarose gel stained with SuperRed/GelRed (BS354B, Biosharp, https://www.biosharp.cn). DNA Gel Extraction Kit (TSP601-50, Tsingke, https://tsingke.com.cn/products/details/149) was used for acquiring the PCR products and followed by Sanger sequencing.

Identify the gene expression level of each sample by RSEM. The clean data generated by Illumina sequencing were mapped to SMRT sequencing data, and the read count of each gene was obtained from the mapping results. The read count value of each gene was converted to the FPKM value (Fragments per Kilobase Million), and genes with FPKM > 0.3 were selected for analysis.

### Statistical analysis

Values of morphological and agronomic characteristics were expressed as means ± standard deviations. Data were analyzed by one-way ANOVA using the SPSS statistical software (version 25.0). *P* < 0.05 was used to denote a statistically significant difference.

### Data availability statement

The SMRT sequencing raw reads reported in this study have been deposited in the NCBI Sequence Read Archive (SRA) under Bioproject accession number PRJNA699388.

## Supplementary Information

**Additional file 1: Fig.** **S1.** Flowchart of the experimental design and analysis for PacBio sequencing and RNA sequencing. **Fig.** **S2 **Read length with different ploidy and genome compositions hybrids. **Fig.** **S3** The number of isoforms of functional annotation of with different ploidy hybrids. **Fig.** **S4** RT-PCR validation of AS events for two genes. Gel bands in each figure show DNA makers and PCR results in five tissues/samples. **Fig. S5** The p-value for GO enrichment of isoforms.

**Additional file 2:** **Table S1.** Functional annotation of transcripts.

**Additional file 3:** **Table S2.** Splice junctions and AS modes with different ploidy levels.

**Additional file 4:** **Table S3.** Functional annotation of transcripts in the polyadenylation events.

**Additional file 5:** **Table S4 **Summary of primers used in this study.

## Data Availability

The data supporting the conclusions of this article are provided within the article and its supplementary information files. The SMRT sequencing raw reads reported in this study have been deposited at NCBI Sequence Read Archive (SRA) under Bioproject accession number PRJNA699388.

## Abbreviations

AS: Alternative splicing
APA: Alternative polyadenylation
IR: Intron retention
A5: Alternative 5' splice site
A3: Alternative 3' splice site
ES: Exon skipping
MX: Mutually exclusive exons
KOG/COG: Cluster of orthologous groups
GO: Gene ontology
KEGG: Kyoto encyclopedia of genes and genomes
