# Supplementary material for: Full-length transcriptome reconstruction reveals genetic differences in hybrids of Oryza sativa and Oryza punctata with different ploidy and genome compositions
Source: BMC Plant Biol. 2022 Mar 21;22:131. doi: 10.1186/s12870-022-03502-2 (PMC8935693; doi:10.1186/s12870-022-03502-2)
Supplement: Supplementary file 1 — Additional file 1: Fig. S1. Flowchart of the experimental design and analysis for PacBio sequencing and RNA sequencing. Fig. S2 Read length with different ploidy and genome compositions hybrids. Fig. S3 The number of isoforms of functional annotation of with different ploidy hybrids. Fig. S4 RT-PCR validation of AS events for two genes. Gel bands in each figure show DNA makers and PCR results in five tissues/samples. Fig. S5 The p-value for GO enrichment of isoforms. [file 12870_2022_3502_MOESM1_ESM.docx]

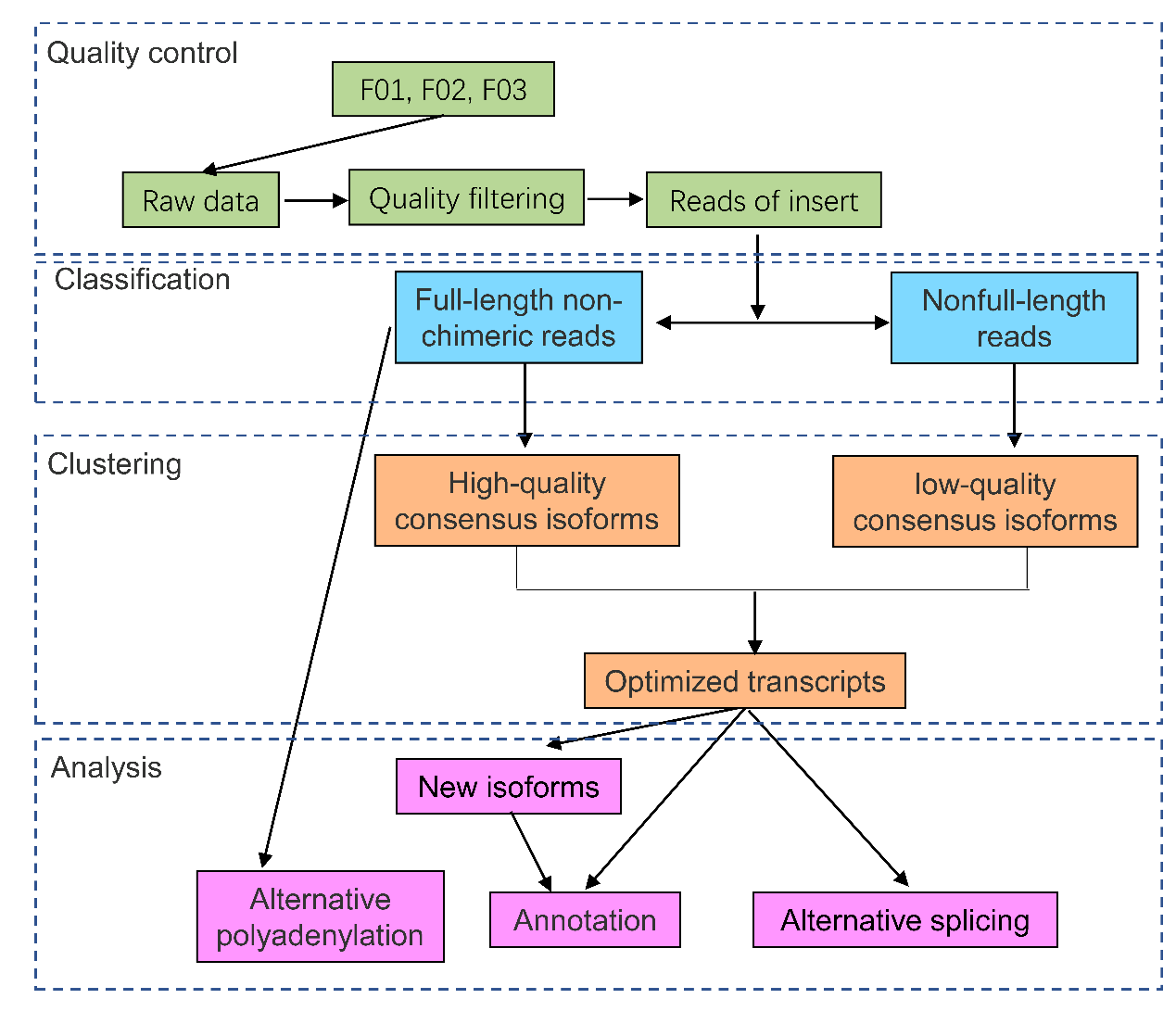


**Fig. S1** Flowchart of the experimental design and analysis for PacBio sequencing and RNA sequencing. This pipeline includes the workflow for the quality control of the raw data, the classification of the reads of the insert, isoform clustering, correction, and transcriptome analysis.


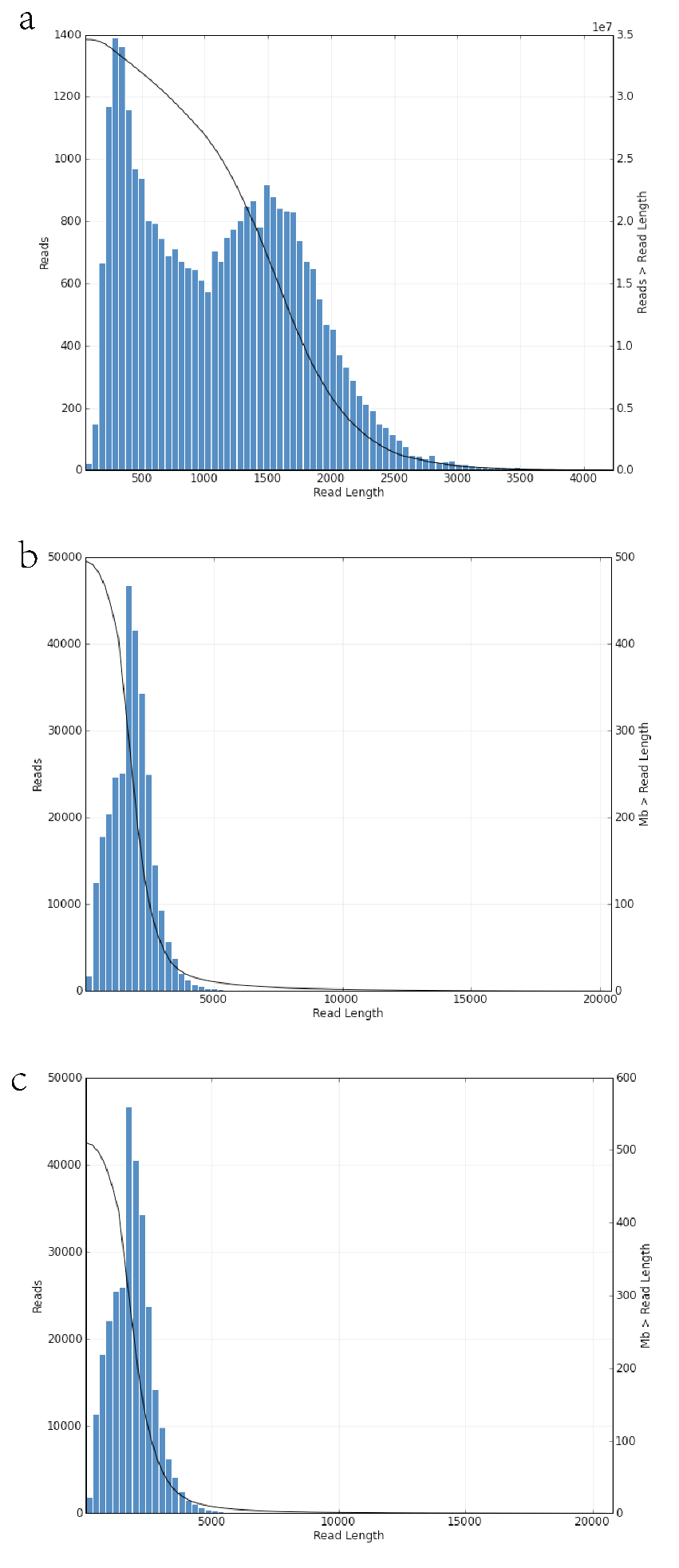


**Fig. S2** Read length with different ploidy and genome compositions hybrids. **a** F01. **b** F02. **c** F03


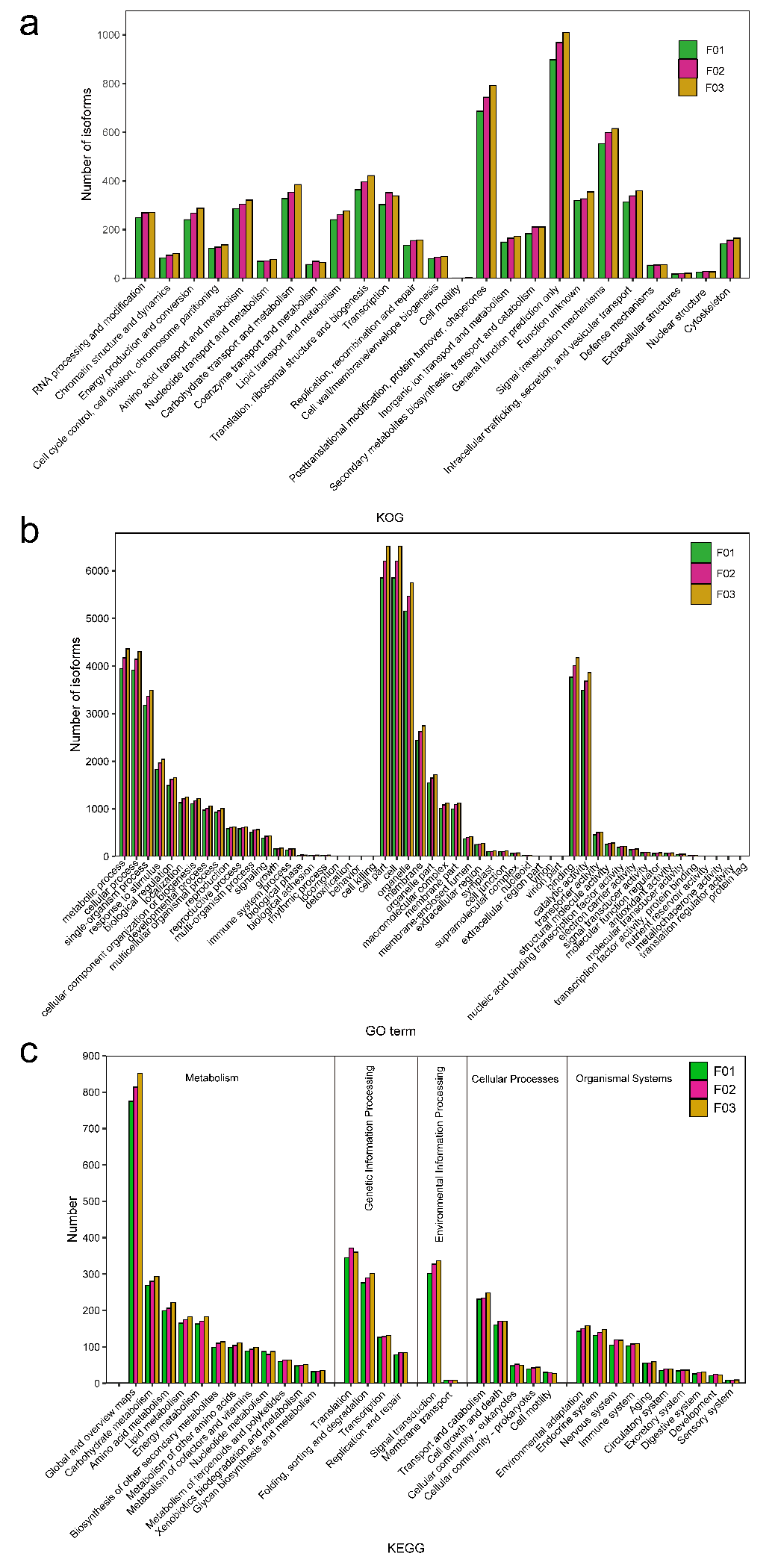


**Fig. S3** The number of isoforms of functional annotation of with different ploidy hybrids. **a** EuKaryotic orthologous groups (KOG) functional classification. **b** gene ontology (GO) enrichment term. **c** Kyoto encyclopedia of genes and genomes (KEGG) enrichment terms.


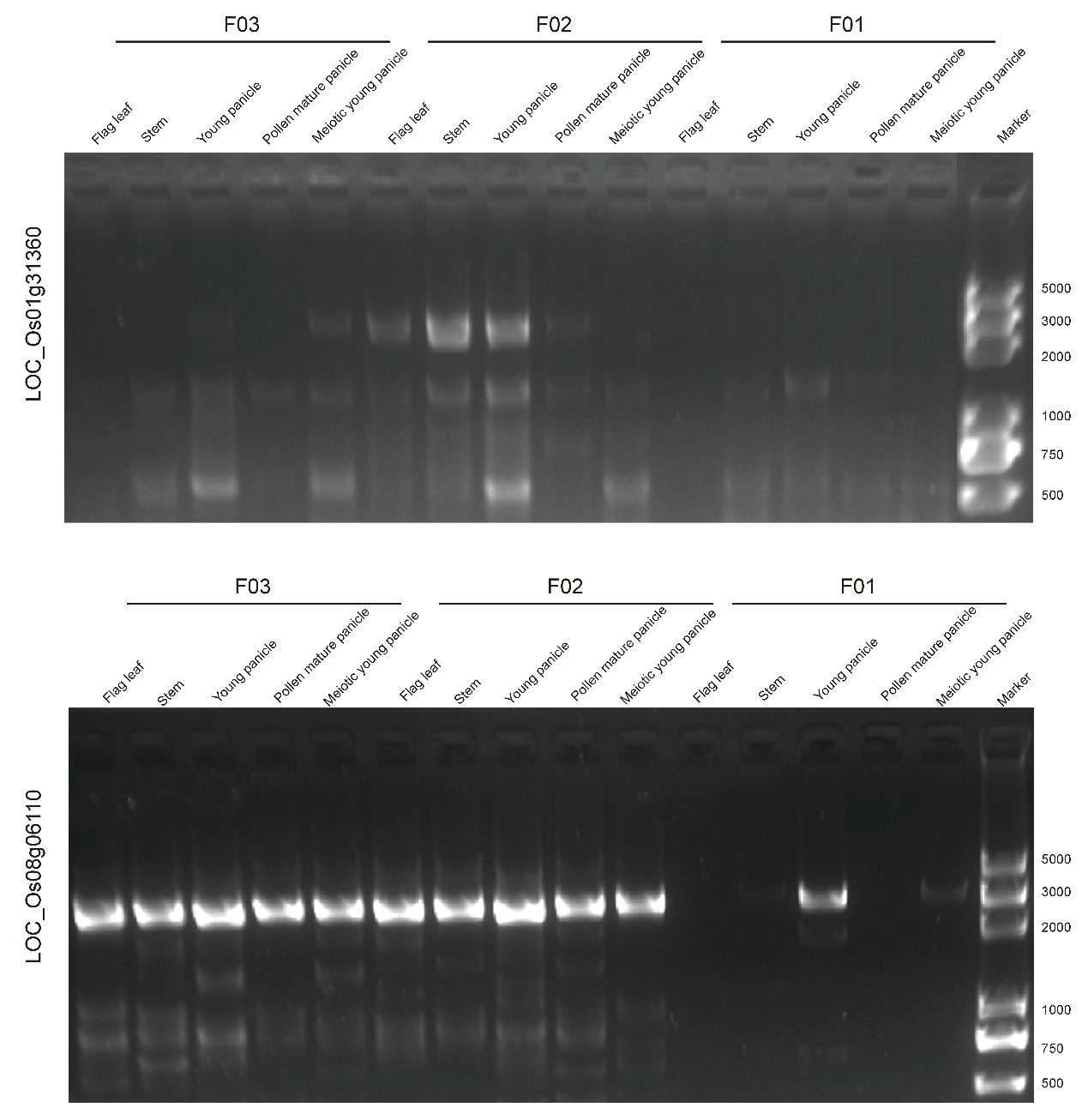


**Fig. S4** RT-PCR validation of AS events for two genes. Gel bands in each figure show DNA makers and PCR results in five tissues/samples.


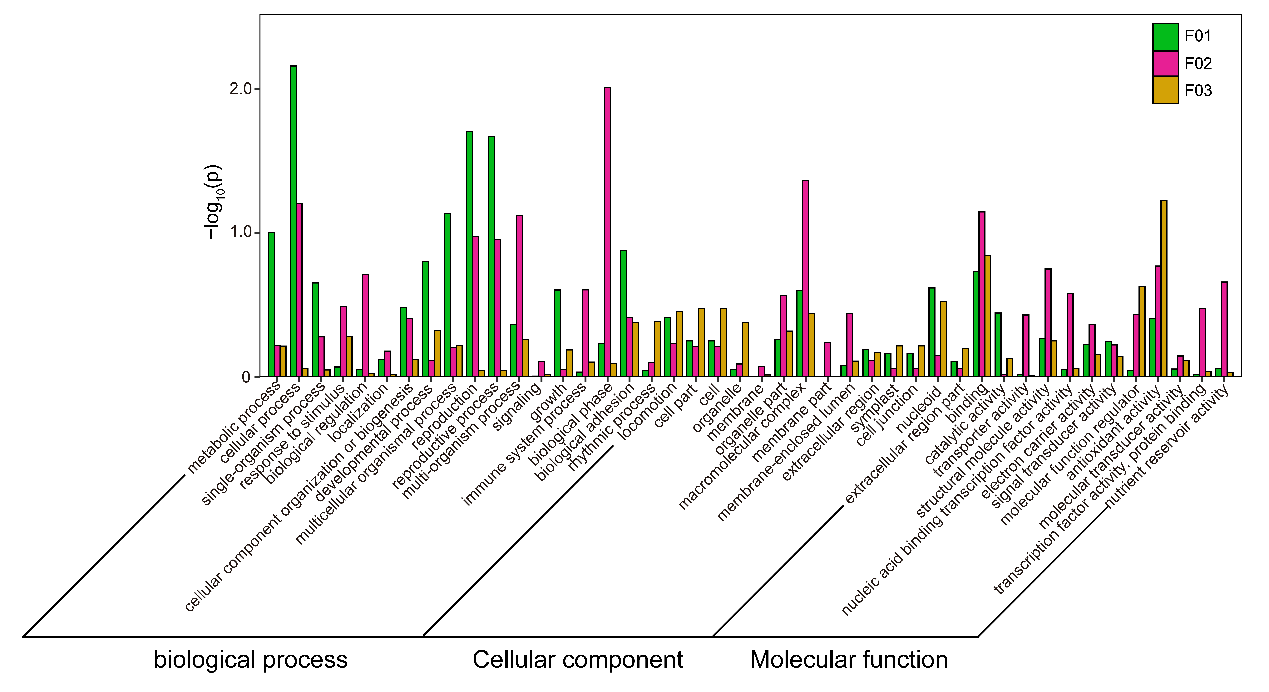


**Fig. S5** The p-value for GO enrichment of isoforms.
