# Supplementary material for: Full-length transcriptome reconstruction reveals genetic differences in hybrids of Oryza sativa and Oryza punctata with different ploidy and genome compositions
Source: BMC Plant Biol. 2022 Mar 21;22:131. doi: 10.1186/s12870-022-03502-2 (PMC8935693; doi:10.1186/s12870-022-03502-2)
Supplement: Supplementary file 5 — Additional file 5: Table S4 Summary of primers used in this study. [file 12870_2022_3502_MOESM5_ESM.docx]

**Table S4** Summary of primers used in this study

| **Gene name** | **Primer sequence** |
| --- | --- |
| LOC_Os08g06110 | F: GGTCTAGGAGGAGGTATGAGGT |
|  | R: GCATTGGAAGGTCTGAAGTC |
| LOC_Os01g31360 | F: CGAGTGGAGGAGGAGGAGAA |
|  | R: ACCAGCCATTCGCAGAGAAA |
| LOC_Os10g38050 | F: CAGTACTGCATGGACACCGT |
|  | R: GAGCTGTCGATCACCCTGAG |
